# Predictors of opinions on prison smoking bans: Analyses of survey data from Scottish staff and prisoners

**DOI:** 10.18332/tid/109559

**Published:** 2019-06-04

**Authors:** Helen Sweeting, Sean Semple, Evangelia Demou, Ashley Brown, Kate Hunt

**Affiliations:** 1 MRC/CSO Social and Public Health Sciences Unit, University of Glasgow, Glasgow, United Kingdom; 2 Institute for Social Marketing, University of Stirling, Stirling, United Kingdom

**Keywords:** smoking restrictions, opinions, secondhand smoke, prisons, policy

## Abstract

**INTRODUCTION:**

Policy-makers and practitioners need to understand characteristics associated with support for smoking restrictions to identify both potential allies and groups requiring particular support/targeted communication in the face of restrictions. Using data from prison staff and prisoners, we explored the structure and correlates of opinions relating to prison smoking bans.

**METHODS:**

Questionnaires were completed by staff (online, N=1271; 27% return) and prisoners (paper-based, N=2512; 34%) in all 15 Scottish prisons in 2016–17. At that time, prisoners could smoke in their own cells and during outdoor recreation; staff smoking was prohibited anywhere on prison grounds. Staff and prisoner questionnaires included identical/very similar questions about opinions on smoking in prisons and prison smoking bans, own smoking behaviour, health and sociodemographic details. We also measured in every prison fine particulate matter (PM_2.5_) as a proxy for secondhand smoke (SHS) levels.

**RESULTS:**

Principal components analysis identified two factors: ‘Positive about bans’ (higher scores among staff) and ‘Bans will be difficult’ (higher scores among prisoners). In multivariable analyses, ‘Positive about bans’ was associated with: not smoking (both staff and prisoners), better general health, more respiratory symptoms and working in an operational role among staff; and no asthma, more sensory symptoms, higher educational level and status/release date among prisoners. ‘Bans will be difficult’ was associated with: fewer sensory symptoms and lower prison SHS levels among staff and being a smoker among prisoners. In smoker-only analyses, heavier smokers were less positive about bans and more likely to believe bans will be difficult.

**CONCLUSIONS:**

Results suggest it is possible to be positive about prison smoking bans whilst also recognising and/or concerned about potential operational difficulties, and that these opinions are associated with several characteristics additional to smoker status. Support for future prison bans may be stronger if staff have access to objective SHS exposure measures.

## INTRODUCTION

Breathing in secondhand tobacco smoke (SHS) harms health, with SHS exposure estimated as causing around 1% of total worldwide mortality^1^. Restrictions to SHS exposure, which are most successfully achieved via smoke-free legislation, have clear health benefits^2^. Support for smoke-free policy is key to successful implementation^3,4^. It is therefore important to identify not only levels of support among those subject to smoking restrictions, but also characteristics associated with different levels of support, since these might enable those involved in implementation/enforcement to identify potential allies^5,6^ and better target measures to address expressed concerns and reduce potential problems.

Smoking restrictions have been increasingly introduced in public places since the 1970s, although with variation among jurisdictions in coverage. In many countries that have introduced workplace restrictions, exemptions occur for workplaces such as prisons, which are also regarded as ‘homes’. In the absence of smoke-free policies, prisoner smoking rates are typically high, around 2–8 times those of the general population in studies internationally^7^. The health implications for prisoners who smoke and those (staff/prisoners) exposed to SHS have contributed to the introduction of increased restrictions or, in some jurisdictions, complete smoking bans in prisons^7^. We have previously documented Scottish prison staff and prisoner opinions on prison smoking bans, using survey and focus group data collected several months before the announcement that Scottish prisons would become smoke-free from November 2018^8^. This companion paper, based on survey data only, examines correlates of those opinions.

Surveys of both general populations and those in specific settings almost all find stronger support for hypothetical, proposed or currently implemented smoking restrictions among non-smokers. Examples within the general population include studies conducted in Australia^9^, Europe^10^, and Jordan^11^. Research within specific populations and/or settings also showing greater support for smoking restrictions among non-smokers is similarly international, including for example: Australian university staff and students^3^, Dutch psychiatric hospital staff^12^, and UK bar workers^13^.

Some studies have also identified other characteristics associated with greater support of smoking restrictions. These include older age^9,11,12^, being female^9^, and higher socioeconomic status^9^. Greater support of restrictions has also been found among those with more knowledge of, or stronger beliefs about, harms associated with smoking and SHS exposure^10,11^. Finally, although one study found little difference in levels of support according to self-rated health^10^, others have found greater support among those reporting that SHS irritates their eyes, that they dislike its smell and that increased restrictions would reduce SHS ‘annoyance’^12^.

Some population-based or workplace-based studies have focused specifically on smokers. These have found more positive opinions about smoking restrictions and, variously, lower nicotine dependence, lighter smoking, fewer perceived smoking-related personal benefits, lower smoker identity, and greater intention to quit^5,6,9,14,15^.

Very few surveys have been conducted on opinions about smoking restrictions in prisons; also potentially relevant are surveys in secure hospitals. An Irish study surveyed 90 prison staff among those from other workplaces, reporting agreement with: ‘Should there be a smoking ban in prisons?’ (41% yes); prohibition of smoking in enclosed areas (79% agreed); and whether a complete ban would ‘create more problems in the prison’ (88% yes)^16^. As with population or other workplace-based studies, this study found greater support for restrictions among non-smokers. Greater support for smoking restrictions among non-smokers has also been found among Vermont prison staff (when there was a ban on indoor smoking among both staff and prisoners)^17^; German prisoners (when smoking was allowed in cells)^18^; Australian high security mental health inpatient facility staff^19^; and UK forensic unit in-patients^20^. The Vermont study also found that support for smoking restrictions on prisoners was stronger among prison staff than among prisoners, and uniformed staff (likely to have more contact with prisoners) were more supportive of continuing to permit prisoners to smoke outdoors than non-uniformed staff^17^. Surveys in a US secure psychiatric unit before and after a total ban also found that at both time-points, staff were more likely than patients to support the ban^21^.

### Aims

This paper, based on survey data from Scottish prison staff and prisoners, aimed to explore:

- the structure of opinions with respect to prison smoking bans (specifically whether factor analysis of several statements about prison smoking bans identified more than one dimension);
- correlates of that/those dimension(s).

## METHODS

### Study design

Data are drawn from the Tobacco In Prisons study (TIPs), which is designed to evaluate the process of implementing enhanced tobacco control in Scottish prisons^8,22.^ TIPs is a three-phase study. Phase 1 was conducted before any announcements about smoking policy changes; Phase 2 following the announcement, but before introduction of a smoke-free policy; and Phase 3 following policy implementation. These Phase 1 survey data were collected in late 2016/early 2017, several months before the (July 2017) announcement that all Scottish prisons would become completely smoke-free from 30 November 2018. The Scottish prison estate consists of 13 publicly and two privately managed prisons. Most hold a mix of offender types and include: 10 accommodating males only, one with females only and four with both males and females, one accommodating male young offenders (aged 16–21 years), and one open prison accommodating low-supervision adult male offenders. At the time of data collection, prisoners were allowed to smoke in their own cells and during outdoor recreation; staff and visitors were prohibited from smoking anywhere on prison grounds.

An online prison staff survey was conducted, open 1 November to 16 December 2016. The survey link and reminders were circulated to our staff contacts in all 15 Scottish prisons, with requests to forward these to other prison (but not NHS/visiting) staff. The prisoner survey was conducted via paper questionnaires between November 2016 and April 2017. In two prisons, TIPs staff were escorted around residential areas, distributed questionnaires to all prisoners who said they were willing to complete one, answered queries and helped with completion if necessary. In a third (the open prison), TIPs staff distributed questionnaires during an evening meal. In the remaining 12 prisons, questionnaires were supplied for prison staff to distribute to every prisoner and collect (in sealed envelopes protecting confidentiality), generally during an overnight lock-up. Staff and prisoner questionnaires included identical or very similar questions around opinions on smoking in prisons and prison smoking bans, own smoking, health and sociodemographic details.

### Participants

Questionnaires were completed by 1271 staff (27%) and 2512 prisoners (34%). Response/return rates varied considerably among prisons: 10–38% for staff, and 10–60% for prisoners. The staff sample was identical with respect to proportions of males (71%) and females (29%), and very similar with respect to age, for Scottish Prison Service staff in post as at 31 March 2017^23^. The prisoner sample included a slightly higher proportion of females (7%) than within the Scottish prison population overall (5%)^23^, and prisoner smoking rates (74%) were slightly higher than those reported in the 2015 (72%) and 2017 (68%) Scottish prisoner surveys^24^.

### Measures

Table 1 shows question wordings and analytic categories (if appropriate) for all variables.

**Table 1:** Dependent and independent variables — exact question wording and analytic categories (if appropriate)

*Dependent variables*	*Question wording*	*Analytic categories*
**Smoking ban opinion items**	You have probably heard that smoking is no longer allowed in any areas (inside and outside) in prisons in some countries around the world, like Canada, New Zealand and Wales. What do you think of prison smoking bans like these? (strongly agree, agree, no opinion, disagree, strongly disagree for all) Prison smoking bans are a good idea Prison smoking bans cause a lot of trouble (e.g. prisoner fights, rioting, tobacco smuggling) Prison smoking bans help prisoners stop smoking long-term (and after release) Prison smoking bans are hard to enforce Most staff want prison smoking bans Prison smoking bans are OK if enough stop smoking support is available to prisoners Prison smoking bans are OK if prisoners are allowed e-cigarettes or vapes
**In favour of increased smoking restrictions**	Would you be in favour of increased smoking restrictions in Scottish prisons? (Yes — I would be in favour of increased smoking restrictions; I would have no opinion about increased smoking restrictions; No — I would be against increased smoking restrictions)
*AMONG ALL*		
**Sex**	Are you male or female?	Male Female
**Age** (years)	STAFF – How old are you? PRISONERS – What is your age?	≤30 31–40 41–50 ≥51
**Current smoker**	STAFF – If yes to ‘Have you ever smoked a cigarette?’: Do you smoke cigarettes nowadays? (yes/no) PRISONERS – If yes to ‘Have you ever smoked a cigarette?’: Do you smoke cigarettes now (in prison)? (yes/no)	Yes No
**General health**	How is your health in general? (very good, good, fair, bad, very bad)	Very good Good Fair, bad or very bad
**Diagnosed asthma**	Has a doctor ever told you that you have asthma? (yes/no)	Yes No
**Respiratory symptoms**	In the past 4 weeks have you: (yes/no, to each) Had a cold? Had wheezing or whistling in your chest? Felt short of breath? Usually coughed first thing in the morning? Coughed at all during the rest of the day or night? Brought up any phlegm?	None 1–2 3–6
**Sensory symptoms**	In the past 4 weeks have: (yes/no, to each) Your eyes been red or irritated? You had a runny nose, sneezing or nose irritation? You had a sore or scratchy throat?	None 1–2 3
**Anxiety or depression** (EQ5D5L)^28^	Please click (STAFF) / tick (PRISONERS) the box that best describes your health today: anxiety/depression (I am not anxious or depressed, I am slightly anxious or depressed, I am moderately anxious or depressed, I am severely anxious or depressed, I am extremely anxious or depressed)	None Slight Moderate, severe or extreme
**Prison SHS** (PM_2.5_ outdoor adjusted)	Area measurement of fine Particulate Matter (PM_2.5_) concentrations (μg/m^3^) over a 6-day period within a hall or landing area in each prison using Dylos DC1700 monitors^22^.	Low (<10, four prisons) Mid (10–39, seven prisons) High (>39, four prisons)
*AMONG STAFF ONLY*		
**Highest educational level**	What was the highest level of education you received? (school, FE college, university)	School Further education Higher education
**Staff role**	Is your role operational or non-operational?	Operational Non-operational
**Staff band**	What is your band? (Scottish Prison Service Band B; privately run prisons Administrative / Scottish Prison Service Bands C+D; privately run prisons Prison Officer /Physical Education Instructor /Scottish Prison Service Bands E+F; privately run prisons First line /Middle manager / Scottish Prison Service Band G; privately run prisons Head of Function /Governor /Director)	SPS B-D (Admin, prison officer, Phys. Ed) SPS E-G (Manager, head of function, governor)
**Staff years worked in prisons**	Altogether, how many years have you worked in prisons in total?	0–1 2–4 5–9 ≥10
*AMONG PRISONERS ONLY*		
**Age left education**	How old were you when you left full-time education?	≤14 15–16 ≥17 years
**Prisoner status**	Are you convicted or on remand? (yes/no) If you are convicted, how long is your present sentence? (up to 90 days, 3–12 months, 1–4 years, 5–10 years, over 10 years, life)	Unconvicted (awaiting trial) Convicted release within 90 days Convicted – release 3–12 months Convicted – release over a year
*AMONG SMOKERS ONLY*		
**Cigarettes per day**	STAFF – About how many cigarettes do you usually smoke on work days? About how many cigarettes do you usually smoke on your days off? (Daily calculated as average on a workday and days off; overall average calculated as [workday×5 + days off×2]/7) PRISONERS – How many cigarettes (including roll-ups) do you usually smoke each day?	≤10 11–20 ≥21
**Craved cigarettes today**	How much have you craved cigarettes today? (not at all, hardly at all, a little, somewhat, quite a bit, a great deal)	Not or hardly at all A little Somewhat, quite a bit or a great deal
**Quit attempt (in prison) in past year**	STAFF – In the last year have you tried any of these things to help you stop smoking? (yes/no, to each) Attending an NHS stop smoking programme Using electronic cigarettes Using NRT (patches, gum or inhaler) Using prescribed Champix/Varenecline or Zyban/Buproprion PRISONERS – have you tried anything to help you give up smoking in the past year? (yes/no, to each) Tried to get a place on a prison stop smoking programme Went to a group stop smoking programme Went to a one-to-one stop smoking programme Used nicotine replacement (e.g. patches, inhaler) Taken prescribed medicine (Champix, Zyban)	Yes No

#### Dependent variables

Prison smoking ban opinion items^9^ were adapted from surveys of US prison staff opinions about restrictions to smoking in prisons^17^ and Scottish bar workers’ attitudes to smoke-free public places legislation^13^. They comprised seven items on whether prison smoking bans: 1) are a good idea, 2) cause a lot of trouble, 3) stop prisoners smoking long-term, 4) are hard to enforce, 5) are OK if stop smoking support available, 6) are OK if e-cigarettes available, and 7) are wanted by most staff; with five answer options and a single item on agreement with increased smoking restrictions in Scottish prisons.

#### Independent variables

Staff and prisoners were asked about their age, sex and education. The questionnaires also asked about: staff role, seniority band and number of years they had worked in prisons, and prisoner status (unconvicted, convicted and time to release).

All participants were asked their current smoking status. Smokers were asked about daily cigarettes smoked, how much they had craved cigarettes today^25^, and whether they had tried a number of quit-smoking strategies in the past year.

All were asked health-related questions. These included health in general using a standard UK survey item^26^: ‘has a doctor ever told you that you have asthma?’; and self-reported past month respiratory (wheezing/whistling, shortness of breath, morning cough, other cough, phlegm) and sensory symptoms (red/irritated eyes, runny nose/sneezing, sore/scratchy throat). The symptom questions were based on International Union Against Tuberculosis and Lung Disease items previously included in studies of bar workers’ health following smoke-free legislation^27^. The questionnaires also included the EQ-5D-5L, a standardised measure of health status including an item on current anxiety/depression^28^.

Between September 2016 and January 2017, TIPs also measured staff and prisoner SHS exposure in all Scottish prisons using multiple methods, including 6-day area measurement of fine Particulate Matter (PM2.5)^22^.

### Ethics

TIPs was approved by the Scottish Prison Service Research Access and Ethics Committee and University of Glasgow’s College of Social Sciences Ethics Committee (ref: 400150214 for staff and prisoner data).

### Analyses

Factor analyses (principal components analysis, varimax rotation) were conducted on the eight prison smoking ban opinion items. Exploratory analyses found one (‘Prison smoking bans are OK if prisoners are allowed e-cigarettes or vapes’) loaded on different factors for staff and prisoners (Supplementary Table 1). Analyses excluding this item resulted in two identical factors for staff and prisoners that explained 16% more total variance than an analysis constrained to a single factor (Supplementary Table 2). Factor analysis was therefore conducted of the remaining seven items for the prisoner and staff groups combined. The two resulting opinion factors (described in Results) were saved and formed the dependent variables in analyses of association.

Staff-prisoner differences on the prison smoking bans opinion factors (F-tests) and all other (independent) variables (chi-squared tests) were examined. Bivariable analyses (F-tests) examined differences in the two opinion factors according to each independent variable, separately for staff and for prisoners. ANOVAs entering the independent variable and one representing staff versus prisoner tested for significant interactions, indicating different associations within staff versus prisoners. Multivariable analyses (SPSS general linear models) entered those independent variables identified via bivariable analyses as significantly related (p<0.05) to each opinion factor, separately for staff and for prisoners. Similar analyses were conducted, restricted to smokers, entering cigarettes per day, craving and past-year quit attempt.

We tested for clustering by introducing a random intercept for prison in two key analyses (models entering staff versus prisoner, and smoker versus non-smoker; Supplementary Table 3). This was nonsignificant, therefore all analyses were conducted via standard linear models. As staff and prisoner questionnaire return rates varied among prisons, simple weights were derived to adjust for this. The results of final multivariable analyses based on unweighted and weighted data (Supplementary Table 4) were virtually identical, so results of unweighted analyses are presented.

## RESULTS

Table 2 shows how each opinion statement loaded on the two opinion factors, for staff, prisoners and both groups combined. The first factor (‘Positive about bans’, 45.8% variance explained) included five items loading 0.6 or greater (prison smoking bans: are a good idea, are OK if stop smoking support available, stop prisoners smoking long-term, are wanted by most staff, and in favour of increased smoking restrictions in Scottish prisons). The second factor (‘Bans will be difficult’, 22.4% variance explained) included two items loading 0.6 or greater (prison smoking bans: are hard to enforce, and cause a lot of trouble).

**Table 2:** Factor analyses of smoking ban items^a^ conducted for staff, prisoners, and both – rotated component matrices for each group, ordered as per ‘staff and prisoners’

	*STAFF*		*PRISONERS*		*STAFF AND PRISONERS*	
	*Positive*	*Difficult*	*Positive*	*Difficult*	*Positive^b^*	*Difficult^c^*
Prison smoking bans are a good idea	**0.823**	-0.307	**0.834**	-0.297	**0.857**	-0.292
Would you be in favour of increased smoking restrictions in Scottish prisons?	**0.800**	-0.170	**0.795**	-0.255	**0.833**	-0.231
Prison smoking bans are OK if enough stop smoking support is available to prisoners	**0.750**	0.197	**0.789**	-0.069	**0.803**	0.006
Prison smoking bans help prisoners stop smoking long-term (and after release)	**0.684**	-0.318	**0.773**	-0.172	**0.784**	-0.221
Most prison staff want smoking bans	**0.730**	-0.341	**0.570**	0.214	**0.639**	-0.093
Prison smoking bans are hard to enforce	-0.125	**0.853**	0.045	**0.840**	-0.024	**0.889**
Prison smoking bans cause trouble (e.g. prisoner fights, rioting, tobacco smuggling)	-0.176	**0.836**	-0.300	**0.737**	-0.332	**0.760**
**Per cent variance explained**	**41.8**	**25.8**	**42.3**	**21.2**	**45.8**	**22.4**

a Item ‘Prison smoking bans are OK if prisoners are allowed e-cigarettes or vapes’ excluded as it loaded on ‘Bans will be difficult’ for staff and ‘Positive about bans’ for prisoners (Supplementary Table 1).

b Saved factor ‘Positive about bans’: range = -2.46 to 2.07, mean = 0.0, SD = 1.0.

c Saved factor ‘Bans will be difficult’: range = -3.28 to 1.65, mean = 0.0, SD = 1.0.

Supplementary Table 5 shows mean scores on the opinion factors and distributions on the independent variables. Staff scores were significantly (p<0.001) higher than those of prisoners on ‘Positive about bans’ (mean factor scores 0.647 and -0.351, respectively), but lower on ‘Bans will be difficult’ (mean scores -0.234 and 0.127, respectively). There were also significant staff-prisoner differences on all independent variables measured across both groups. Prisoners were more likely to be male and younger. Prisoners were also much more likely than staff to be smokers (74.2% vs 9.7%) and, among smokers, prisoners were heavier smokers, reported more craving and were less likely to have made a past-year quit attempt. Prisoners were more likely to report doctor-diagnosed asthma, more respiratory symptoms, fewer sensory symptoms and much higher rates of anxiety/depression. While similar proportions of staff and prisoners worked/lived in one of the prisons with lower measured SHS levels, staff were more likely than prisoners to be in one with higher levels. Most staff respondents had school-leaving or further educational levels and worked in operational and non-managerial roles. Most prisoners had left education by the age of 16 years and were convicted with three months or (much) longer until release.

Table 3 shows non-smokers scored higher on the ‘Positive about bans’ factor among both staff and, particularly, prisoners. Among both, those reporting the best health, but also those reporting more sensory symptoms had higher ‘Positive about bans’ scores. Among staff, those reporting more respiratory symptoms were also more positive about prison smoking bans, while among prisoners, those reporting no asthma were more positive. Higher ‘Positive about bans’ scores were also seen for older prisoners and staff working in prisons with higher measured SHS; neither sex nor anxiety/depression were associated with ‘Positive about bans’ in either group. In staff-only analyses, those in operational roles had higher ‘Positive about bans’ scores. Among prisoners, those with more education and those with longer time until release were more positive, while unconvicted prisoners were least positive. Finally, among smokers only, those reporting lower cigarette consumption (both groups) and prisoners with fewer cravings and a past-year quit attempt were more positive about prison smoking bans.

**Table 3:** Positive about bans factor according to independent variables – bivariable associations for staff and for prisoners, and significance of staff-by-prisoner interaction

	*STAFF*		*PRISONERS*		*STAFF × PRISONER (sig)*
	*Mean*	*F (sig)*	*Mean*	*F (sig)*	
*AMONG ALL*					
**Sex**					
Male	0.665		-0.347		
Female	0.636	0.4 (0.552)	-0.410	0.7 (0.413)	(0.712)
**Age** (years)					
≤30	0.702		-0.475		
31–40	0.708		-0.375		
41–50	0.633		-0.321		
≥51	0.620	1.0 (0.391)	-0.077	15.4 (<0.001)	(<0.001)
**Current smoker**					
Yes	-0.101		-0.621		
No	0.727	140.3 (<0.001)	0.402	700.2 (<0.001)	(0.021)
**General health**					
Very good	0.729		-0.238		
Good	0.619		-0.333		
Fair, bad or very bad	0.541	4.4 (0.013)	-0.427	6.4 (0.002)	(0.845)
**Diagnosed asthma**					
Yes	0.678		-0.439		
No	0.634	0.6 (0.424)	-0.323	6.5 (0.011)	(0.034)
**Respiratory symptoms**					
None	0.498		-0.283		
1–2	0.643		-0.392		
3–6	0.787	13.3 (<0.001)	-0.376	2.8 (0.060)	(0.002)
**Sensory symptoms**					
None	0.470		-0.480		
1–2	0.679		-0.257		
3	0.875	22.6 (<0.001)	-0.154	20.8 (<0.001)	(0.843)
**Anxiety or depression** (EQ5D)					
None	0.630		-0.321		
Slight	0.704		-0.335		
Moderate, severe or extreme	0.639	0.9 (0.407)	-0.390	1.3 (0.262)	(0.455)
**Prison SHS** (PM_2.5_ outdoor adjusted)					
Prison with low SHS (<10)	0.606		-0.350		
Prison with mid SHS (10–39)	0.606		-0.355		
Prison with high SHS (>39 μg/m^3^)	0.755	4.7 (0.009)	-0.344	0.0 (0.973)	(0.142)
*AMONG STAFF ONLY*					
**Highest educational level**					
School	0.676				
Further education	0.624				
Higher education	0.651	0.5 (0.582)			n/a
**Staff role**					
Operational	0.676				
Non-operational	0.520	7.7 (0.006)			n/a
**Staff band**					
SPS B-D (Admin, prison officer, Phys. Ed)	0.669				
SPS E-G (Manager, head of function, governor)	0.593	1.6 (0.197)			n/a
**Staff years worked in prisons**					
0–1	0.572				
2–4	0.652				
5–9	0.677				
≥10	0.649	0.4 (0.788)			n/a
*AMONG PRISONERS ONLY*					
**Age left education**					
≤14			-0.560		
15–16			-0.383		
≥17 years			-0.127	23.3 (<0.001)	n/a
**Prisoner status**					
Unconvicted (awaiting trial)			-0.518		
Convicted – release within 90 days			-0.469		
Convicted – release within 3–12 months			-0.413		
Convicted – release over a year			-0.182	16.7 (<0.001)	n/a
*AMONG SMOKERS ONLY*					
**Cigarettes per day**					
≤10	0.088		-0.438		
11–20	-0.222		-0.632		
≥21	-0.593	3.5 (0.034)	-0.724	11.0 (<0.001)	(0.359)
**Craved cigarettes today**					
Not or hardly at all	-0.015		-0.474		
A little	0.019		-0.494		
Somewhat, quite a bit or a great deal	-0.266	1.2 (0.305)	-0.697	13.8 (<0.001)	(0.926)
**Quit attempt (in prison) in past year**					
Yes	-0.080		-0.372		
No	-0.200	0.4 (0.522)	-0.813	127.5 (<0.001)	(0.057)

Table 4 shows that among both groups, younger people and smokers had higher ‘Bans will be difficult’ factor scores. Among staff, those reporting worse health, but also those reporting no respiratory or sensory symptoms were more likely to believe bans will be difficult. Prisoners reporting greater anxiety/depression and staff in prisons with lower measured SHS had higher scores on this factor. Higher ‘Bans will be difficult’ scores occurred among staff with least experience of working in prisons and prisoners with least education and those who were unconvicted. In smoker-only analyses, prisoners with greater cravings, but also those with a past-year quit attempt, had higher ‘Bans will be difficult’ scores.

**Table 4:** Bans will be difficult factor according to independent variables – bivariable associations for staff and for prisoners, and significance of staff-by-prisoner interaction

	*STAFF*		*PRISONERS*		*STAFF × PRISONER (sig)*
	*Mean*	*F (sig)*	*Mean*	*F (sig)*	
*AMONG ALL*					
**Sex**					
Male	-0.266		0.125		
Female	-0.137	3.6 (0.058)	0.161	0.2 (0.641)	(0.364)
**Age** (years)					
≤30	-0.027		0.221		
31–40	-0.278		0.131		
41–50	-0.304		0.066		
≥51	-0.249	3.2 (0.023)	0.027	4.5 (0.003)	(0.370)
**Current smoker**					
Yes	0.065		0.218		
No	-0.265	10.5 (0.001)	-0.132	63.8 (<0.001)	(0.850)
*AMONG ALL*					
**General health**					
Very good	-0.334		0.059		
Good	-0.185		0.144		
Fair, bad or very bad	-0.158	3.0 (0.049)	0.146	1.5 (0.215)	(0.449)
**Diagnosed asthma**					
Yes	-0.336		0.149		
No	-0.212	2.7 (0.101)	0.129	0.2 (0.664)	(0.089)
**Respiratory symptoms**					
None	-0.160		0.094		
1–2	-0.142		0.150		
3–6	-0.359	4.9 (0.008)	0.155	1.0 (0.379)	(0.667)
**Sensory symptoms**					
None	-0.087		0.131		
1–2	-0.224		0.156		
3	-0.537	13.7 (<0.001)	0.067	0.9 (0.402)	(0.029)
**Anxiety or depression** (EQ5D)					
None	-0.216		0.040		
Slight	-0.262		0.098		
Moderate, severe or extreme	-0.269	0.3 (0.748)	0.209	7.5 (0.001)	(0.059)
**Prison SHS** (PM_2.5_ outdoor adjusted)					
Prison with low SHS (<10)	-0.108		0.103		
Prison with mid SHS (10–39)	-0.171		0.161		
Prison with high SHS (>39 μg/m^3^)	-0.455	10.9 (0.000)	0.075	1.8 (0.162)	(0.003)
*AMONG STAFF ONLY*					
**Highest educational level**					
School	-0.266				
Further education	-0.192				
Higher education	-0.249	0.6 (0.539)			n/a
**Staff role**					
Operational	-0.242				
Non-operational	-0.198	0.3 (0.576)			n/a
**Staff band**					
SPS B-D (Admin, prison officer, Phys. Ed)	-0.259				
SPS E-G (Manager, head of function, governor)	-0.154	1.6 (0.199)			n/a
**Staff years worked in prisons**					
0–1	-0.066				
2–4	-0.150				
5–9	-0.114				
≥10	-0.303	2.9 (0.033)			n/a
*AMONG PRISONERS ONLY*					
**Age left education**					
≤14			0.203		
15–16			0.153		
≥17 years			0.032	4.2 (0.015)	n/a
*AMONG PRISONERS ONLY*					
**Prisoner status**					
Unconvicted (awaiting trial)			0.247		
Convicted – release within 90 days			0.073		
Convicted – release within 3–12 months			0.146		
Convicted – release over a year			0.103	2.8 (0.040)	n/a
*AMONG SMOKERS ONLY*					
**Cigarettes per day**					
≤10	0.062		0.122		
11–20	0.185		0.238		
≥21	-0.545	2.5 (0.085)	0.270	2.8 (0.064)	(0.035)
**Craved cigarettes today**					
Not or hardly at all	-0.136		0.146		
A little	0.141		0.118		
Somewhat, quite a bit or a great deal	0.198	1.5 (0.237)	0.283	6.4 (0.002)	(0.329)
**Quit attempt (in prison) in past year**					
Yes	0.048		0.268		
No	0.142	0.2 (0.641)	0.181	4.1 (0.044)	(0.331)

In multivariable analyses, ‘Positive about bans’ factor scores remained significantly associated with not smoking for both staff and prisoners; with better general health, more respiratory symptoms and working in an operational role among staff; and with no asthma, more sensory symptoms, higher educational level and status (unconvicted versus convicted and release date) among prisoners (Table 5). Variance explained was much higher with respect to prisoners, attributable to the very strong association between own smoking and ‘Positive about bans’.

**Table 5:** Bans factors according to independent variables – multivariable associations for staff and for prisoners

	*STAFF*		*PRISONERS*	
	*F*	*(sig)*	*F*	*(sig)*
*AMONG ALL*				
**Positive about bans factor**				
Population: STAFF 1108, PRISONERS 2011				
Age	-	-	0.7	(0.578)
Current smoker	120.7	(<0.001)	460.9	(<0.001)
General health	5.4	(0.005)	0.0	(0.977)
Diagnosed asthma	-	-	4.5	(0.033)
*AMONG ALL*				
**Positive about bans factor**				
Population: STAFF 1108, PRISONERS 2011				
Respiratory symptoms	9.1	(<0.001)	-	-
Sensory symptoms	3.0	(0.051)	25.2	(<0.001)
Prison SHS (PM_2.5_ outdoor adjusted)	2.7	(0.065)	-	-
Staff – role	4.1	(0.044)	n/a	
Prisoner – age left education	n/a		5.4	(0.004)
Prisoner – unconvicted /convicted /release status	n/a		4.8	(0.003)
(Adjusted R^2^)	(0.148)		(0.254)	
**Bans will be difficult factor**				
Population: STAFF 1085, PRISONERS 2077				
Age	1.0	(0.386)	2.0	(0.109)
Current smoker	3.4	(0.065)	35.9	(<0.001)
General health	2.5	(0.085)	-	-
Respiratory symptoms	1.6	(0.203)	-	-
Sensory symptoms	6.3	(0.002)	-	-
Anxiety or depression (EQ5D)	-	-	2.5	(0.081)
Prison SHS (PM_2.5_ outdoor adjusted)	5.8	(0.003)	-	-
Staff – years worked in prisons	0.5	(0.713)	n/a	
Prisoner – age left education	n/a		1.2	(0.292)
Prisoner – unconvicted /convicted /release status	n/a		2.1	(0.100)
(Adjusted R^2^)	(0.039)		(0.030)	
*AMONG SMOKERS ONLY*				
**Positive about bans factor**				
Population: PRISONERS 1596				
Cigarettes per day	-	-	7.1	(0.001)
Craved cigarettes today	-	-	7.5	(0.001)
Quit attempt (in prison) in past year	-	-	132.4	(<0.001)
(Adjusted R^2^)			(0.094)	
**Bans will be difficult factor**				
Population: PRISONERS 1614				
Craved cigarettes today	-	-	8.4	(<0.001)
Quit attempt (in prison) in past year	-	-	4.4	(0.037)
(Adjusted R^2^)			(0.011)	

Fewer variables were independently associated with ‘Bans will be difficult’, and variance explained was much lower. This was particularly the case for prisoners, where only current smoking was significantly positively associated in the multivariable model. Among staff, fewer sensory symptoms and lower prison SHS levels, but not own smoking, were independently associated with ‘Bans will be difficult’.

Finally, multivariable analyses restricted to prisoners who smoked (no equivalent analyses for staff since multiple variables were not significantly associated with either opinion factor among staff smokers) showed that all variables significant in bivariable analyses of association remained so in the multivariable analyses. Thus, among prisoners who smoked, cigarettes per day remained inversely associated with ‘Positive about bans’, and craved cigarettes and past-year quit attempt remained associated with both opinions factors.

Given staff versus prisoners and smoker versus non-smoker differences in scores on the two smoking ban opinion factors, additional analyses (not shown in Tables) were conducted to see if the much higher prisoner smoking rate could account for differences in staff and prisoner opinions. Whilst the differences decreased substantially after adjustment for smoking status, they remained significant: ‘Bans are positive’ according to staff/prisoner F=1050.6 (p<0.001) before, and F=110.8 (p<0.001) after adjustment for smoking status; whilst ‘Bans will be difficult’ according to staff/prisoner F=108.8 (p<0.001) before, and F=10.1 (p=0.002) after adjustment.

## DISCUSSION

Analyses of survey responses from Scottish prison staff and prisoners on items eliciting opinions about prison smoking bans, collected before announcements about a smoke-free policy implementation date in all Scottish prisons, suggest opinions in both groups could be related to two underlying dimensions (factors). The first, accounting for most variance, represented ‘positive’ opinions towards prison smoking bans, and the second highlighted their potential difficulties. Importantly, this suggests it is possible to be both generally positive about prison smoking bans, whilst also recognising (and potentially concerned about) the operational difficulties they may bring. This was also evident in analyses of qualitative data from Scottish prison staff obtained around the same time that found ‘Staff views were influenced by beliefs about: acceptability of the policy in principle and whether/how bans could be achieved’^8^.

Consistent with almost every other study within both general^9-11^, and specific populations or settings^3,12,13^, including prisons and forensic psychiatric units^16-20^, non-smokers were more likely to be positive about prison smoking bans. They were also less likely to suggest their introduction would bring difficulties. The effects of smoker status were particularly marked for prisoners, probably because staff were not allowed to smoke on prison premises at the time of the survey. This in turn would mean staff smokers, while potentially more sympathetic than non-smokers towards smoking prisoners, had little to lose from a smoking ban (and potentially gains in terms of their own health, reduced temptations to smoke and perceptions of unfairness from seeing prisoners smoking). Also consistent with studies of similar institutions^17,21^, staff were more positive about bans, and less likely to anticipate difficulties than prisoners. Many, but not all, of the staff and prisoner opinion differences were explained by prisoners’ higher smoking rates.

Previous studies have identified a range of other characteristics associated with greater support for smoking bans (in a range of contexts), including: older age^9,11,12^; being female^9^; having higher socioeconomic status^9^; dislike of, or symptoms associated with, SHS^12^; and, among smokers, variables relating to heaviness/dependence and intention to quit^5,6,9,14,15^. Although our analyses did not find differences between the opinions of males and females, there were differences according to age (older prisoners more positive; younger staff and prisoners more likely to identify potential difficulties) and, among prisoners, educational level (those with least education were least positive and most likely to identify potential difficulties). Although there were no differences according to staff band (managerial/non-managerial), those in operational roles (so more likely to have regular exposure to prisoners’ SHS) were more positive about prison smoking bans, and those who had worked for less time in prisons (i.e. less experienced) were more likely to suggest bans would bring operational difficulties. Unconvicted prisoners were least positive about prison smoking bans and most likely to believe they would cause difficulties, while those with the longest time to release were most positive. Given that, in the absence of smoking bans, smoking is a strong part of prison culture^29^, this finding is perhaps unexpected, but may be because those who are unconvicted have little incentive to ‘buy into’ smoking restrictions and/or are concerned about measures further restricting their freedoms. Among staff, but not prisoners, those in prisons where our objective SHS measurements showed higher SHS exposure levels^22^, were more positive about bans and less likely to believe they would cause difficulties. Although staff completed the survey before our SHS results were available, those working in prisons where levels were higher may have inferred this from their own experience and knowledge of their prison’s architecture and ventilation systems.

Few studies have examined associations between health and opinions about smoking restrictions. Among both staff and prisoners, those reporting better general health were more positive about a ban, while staff reporting better health were least likely to say bans will be difficult, and prisoners with no asthma were more positive. Those already in poor health may disregard ‘additional’ health harms or, perhaps, have a more negative outlook generally, including in respect to a ban. However, some of these differences disappeared in the multivariable analyses, suggesting that they were partially accounted for by (non)smoker status. Importantly, experience of sensory symptoms (red/irritated eyes, runny nose/sneezing, sore/scratchy throat) associated with SHS exposure, remained significant in multivariable analyses, suggesting that they may play an important role in reminding people of ongoing exposure to a potential health hazard.

The results of analyses restricted to smokers were also largely consistent with previous studies of attitudes towards smoking restrictions in other populations that have found more positive opinions about smoking restrictions among lighter, less dependent and/or committed smokers^5,6,9,14,15.^ They are also in line with evidence that more nicotine dependent prisoners are a group that might be more likely to try to continue smoking after a prison smoking ban^30^. Heavier, more dependent smokers, without a recent quit attempt (potentially representing more committed smokers) were less positive about prison smoking bans. These associations were generally significant for prisoners, and in the same direction for staff, among whom numbers of smokers were relatively small. More dependent smokers and those reporting a recent quit attempt were also more likely to view bans as bringing operational difficulties. Although counter-intuitive, perhaps the latter group were reflecting on their own difficulties in quitting.

This study has both strengths and limitations. Strengths include its size and the fact that it encompasses both staff and prisoner views collected, for the first time, across all prisons within a country’s criminal justice system. Another major strength is the fact that identification of two opinion dimensions in these quantitative analyses is consistent with the results of our analyses of independent qualitative data from prison staff on smoking bans in prisons. An important limitation is the relatively low response/return rates and potentially unrepresentative samples. However, those included were very similar to all Scottish prison staff and prisoners with respect to characteristics on which comparable data are available. Another limitation is the cross-sectional nature of the self-report data, hence we cannot draw conclusions on causality, particularly on relationships between the health-related variables and opinions on prison smoking bans. Here, the relative lack of association between anxiety/depression and opinions provides some reassurance that the obtained associations were not simply the result of negative affectivity.

Previous studies have highlighted the importance of understanding characteristics associated with different levels of support for smoking restrictions as a way for policy-makers and practitioners to identify potential allies when introducing restrictions^5,6^, and to identify groups who may need particular support or more targeted communication in the face of restrictions.

Our analyses suggest that although smoker status and, among smokers, dependency and smoker identity are significant, the presence of sensory symptoms related to SHS exposure and working in environments where people are more heavily exposed to SHS were determinants of levels of support for smoking restrictions. The latter is a key finding, suggesting that future prison (or other workplace) bans internationally might be more strongly supported by staff if objective measurements of SHS exposure are available; such measurements could be a routine first step in the process towards smoke-free prisons. Identification of two opinion dimensions in our analysis, with the suggestion that it is possible to be both generally positive about prison smoking bans and concerned about potential associated operational difficulties, also has practical implications. It indicates that those who raise concerns over practical issues about implementation/enforcement of prison smoking bans are not necessarily unsupportive of their introduction and, equally, those who express strong support for prison smoking bans may, nevertheless, have some apprehension over unintended negative consequences. While this may be particularly important in respect to prison smoking bans where the operational difficulties are potentially very significant, it suggests policy-makers and implementers need to acknowledge the legitimacy of both dimensions of opinion in their measures to address concerns and mitigate potential problems.

## CONCLUSIONS

Policy-makers and practitioners need to identify potential allies and adopt appropriate communication strategies when introducing smoking restrictions. Those operating within prison services would benefit from recognising the complexity of staff and prisoner opinions about changes to smoking rules in their communication strategy, since those who appear generally positive about prison smoking bans may also be concerned about associated potential operational difficulties. Associations between opinions and characteristics, including both symptoms related to SHS exposure and measured SHS levels, suggest support for future smoking restrictions in prisons (or other workplaces) may be stronger if links with symptoms are highlighted and objective SHS exposure measures included in the communication strategy.
