# Supplementary material for: Predictors of opinions on prison smoking bans: Analyses of survey data from Scottish staff and prisoners
Source: Tob Induc Dis. 2019 Jun 4;17:47. doi: 10.18332/tid/109559 (PMC6662777; doi:10.18332/tid/109559)
Supplement: Supplementary file 1 [file TID-17-47-s1.pdf]

Supplementary Table 1: Factor analyses of smoking ban items conducted for staff, prisoners and both - rotated component matrices for each group, ordered as per 'both staff and prisoners' – showing that Item 'Prison smoking bans are OK if prisoners are allowed e-cigarettes or vapes' loads on 'Bans will be difficult' for staff and 'Positive about bans' for prisoners

	Staff		Prisoners	
	Positive	Difficult	Positive	Difficult
Prison smoking bans are a good idea	**.845**	-.210	**.758**	-.431
Would you be in favour of increased smoking restrictions in Scottish prisons?	**.799**	-.073	**.726**	-.384
Most prison staff want smoking bans	**.771**	-.230	**.510**	.042
Prison smoking bans help prisoners stop smoking long-term	**.733**	-.182	**.731**	-.276
Prison smoking bans are OK if enough stop smoking support is available to prisoners	**.709**	.360	**.841**	-.070
Prison smoking bans are hard to enforce	-.312	**.725**	.080	**.766**
Prison smoking bans are OK if prisoners are allowed e-cigarettes or vapes	.212	**.694**	**.673**	.290
Prison smoking bans cause trouble (eg prisoner fights, rioting, tobacco smuggling)	-.364	**.683**	-.223	**.765**
% variance explained	***40.8***	***21.7***	***38.9***	***20.9***

Supplementary Table 2: Factor analyses of smoking ban items conducted for staff and prisoners combined – showing that two factors (as shown in main paper Table 1) explain 16% more total variance than analysis constrained to a single factor.

	Two factors		Single factor
	Positive	Difficult	
Prison smoking bans are a good idea	**.857**	-.292	.902
Would you be in favour of increased smoking restrictions in Scottish prisons?	**.833**	-.231	.854
Prison smoking bans are OK if enough stop smoking support is available to prisoners	**.803**	.006	.728
Prison smoking bans help prisoners stop smoking long-term (and after release)	**.784**	-.221	.805
Most prison staff want smoking bans	**.639**	-.093	.620
Prison smoking bans are hard to enforce	-.024	**.889**	-.391
Prison smoking bans cause trouble (eg prisoner fights, rioting, tobacco smuggling)	-.332	**.760**	-.618
% variance explained	***45.8***	***22.4***	***52.0***

Supplementary Table 3: Variance component in opinion factors due to prison (Wald z and sig) in models including (a) staff versus prisoners and (b) smoker versus non-smoker

	Variance component due to prison	
	**Wald Z**	***(sig)***
**'Bans are positive'**		
Including fixed effect for staff Vs prisoner	1.923	*(0.054)*
Including fixed effect for smoker Vs non-smoker	1.046	*(0.296)*
**'Bans will be difficult'**		
Including fixed effect for staff Vs prisoner	1.662	*(0.097)*
Including fixed effect for smoker Vs non-smoker	1.562	*(0.118)*

Supplementary Table 4: Bans factors according to independent variables – multivariable associations for staff and for prisoners – ANALYSES OF DATA WEIGHTED TO CORRECT FOR BETWEEN-PRISON DIFFERENCES IN RESPONSE/RETURN RATES.

	Staff		Prisoner	
	F	(Sig)	F	(Sig)
**AMONG ALL**				
Positive about bans factor				
(N)	*(1108)*		*(2011)*	
**Age**	-	-	1.3	(0.256)
**Current smoker**	116.4	(<0.001)	441.2	(<0.000)
**General health**	2.8	(0.062)	0.0	(0.984)
**Diagnosed asthma**	-	-	2.3	(0.131)
**Respiratory symptoms**	8.6	(<0.001)	-	-
**Sensory symptoms**	2.4	(0.094)	26.1	(<0.001)
**Prison SHS (Pm 2.5 outdoor adjusted)**	2.8	(0.059)	-	-
**Staff - role**	3.3	(0.071)	n/a	
**Prisoner - age left education**	n/a		5.8	(0.003)
**Prisoner – unconvicted/convicted/release status**	n/a		4.8	(0.003)
(Adjusted R^2)	*(0.136)*		*(0.246)*	
Bans will be difficult factor				
(N)	*(1085)*		*(2077)*	
**Age**	1.0	(0.409)	2.0	(0.113)
**Current smoker**	3.2	(0.074)	43.4	(<0.001)
**General health**	3.0	(0.049)	-	-
**Respiratory symptoms**	1.0	(0.383)	-	-
**Sensory symptoms**	3.8	(0.023)	-	-
**Anxiety or depression (EQ5D)**	-	-	2.9	(0.053)
**Prison SHS (Pm 2.5 outdoor adjusted)**	6.6	(0.001)	-	-
**Staff – years worked in prisons**	0.7	(0.526)	n/a	
**Prisoner - age left education**	n/a		0.8	(0.441)
**Prisoner – unconvicted/convicted/release status**	n/a		1.4	(0.231)
(Adjusted R^2)	*(0.031)*		*(0.034)*	
**AMONG SMOKERS ONLY**				
Positive about bans factor				
(N)			*(1596)*	
**Cigarettes per day**	-	-	6.6	(0.001)
**Craved cigarettes today**	-	-	9.2	(<0.001)
**Quit attempt in (in prison) in past year**	-	-	130.6	(<0.001)
(Adjusted R^2)			*(0.097)*	
Bans will be difficult factor				
(N)			*(1614)*	
**Craved cigarettes today**	-	-	8.0	(<0.001)
**Quit attempt in (in prison) in past year**	-	-	4.4	(0.036)
(Adjusted R^2)			*(0.010)*	

Supplementary Table 5: Bans factors (mean and standard deviation) and independent variables (N and column percent) with significance of staff-prisoner difference if applicable.

	Staff		Prisoners		
	**Mean**	***(SD)***	**Mean**	***(SD)***	**(F sig)**
Positive about bans factor	0.647	*(0.771)*	-0.351	*(0.932)*	(<0.001)
Bans will be difficult factor	-0.234	*(1.070)*	0.127	*(0.936)*	(<0.001)
	**N**	***(%)***	**N**	***%***	**(Chi-sq sig)**
**Sex**					
Male	901	*(72.3)*	2321	*(93.1)*	
Female	345	*(27.7)*	173	*(6.9)*	(<0.001)
**Age**					
30 years or younger	203	*(16.4)*	765	*(31.7)*	
31-40	232	*(18.7)*	778	*(32.2)*	
41-50	370	*(29.8)*	494	*(20.4)*	
51 or older	435	*(35.1)*	380	*(15.7)*	(<0.001)
**Current smoker**					
Yes	123	*(9.7)*	1858	*(74.2)*	
No	1148	*(90.3)*	647	*(25.8)*	(<0.001)
**General health**					
Very good	443	*(34.9)*	481	*(19.4)*	
Good	671	*(52.8)*	1012	*(40.9)*	
Fair, bad or very bad	157	*(12.4)*	982	*(39.7)*	(<0.001)
**Doctor diagnosed asthma**					
Yes	252	*(20.1)*	587	*(24.6)*	
No	1003	*(79.9)*	1801	*(75.4)*	(0.002)
**Respiratory symptoms**					
None	403	*(35.4)*	779	*(33.2)*	
1-2	380	*(33.4)*	611	*(26.0)*	
3-5	356	*(31.3)*	959	*(40.8)*	(<0.001)
**Sensory symptoms**					
None	416	*(33.3)*	1120	*(46.8)*	
1-2	592	*(47.3)*	1005	*(42.0)*	
3	243	*(19.4)*	267	*(11.2)*	(<0.001)
**Anxiety or depression (EQ5D)**					
None	834	*(66.0)*	853	*(35.4)*	
Slight	260	*(20.6)*	492	*(20.4)*	
Moderate, severe or extreme	169	*(13.4)*	1068	*(44.3)*	(<0.001)
**Prison SHS (Pm 2.5 outdoor adjusted)**					
Work/live in a prison with lower SHS (below 10)	327	*(25.7)*	645	*(25.7)*	
Work/live in a prison with mid SHS (10-39)	591	*(46.5)*	1304	*(51.9)*	
Work/live in a prison with higher SHS (over 39)	353	*(27.8)*	563	*(22.4)*	(0.001)
**AMONG STAFF ONLY**					
**Highest educational level**					
School	445	*(35.2)*			
Further education	519	*(41.1)*			
Higher education	300	*(23.7)*			n/a
**Staff Role**					
Operational	1040	*(82.1)*			
Non-operational	226	*(17.9)*			n/a
**Staff Band**					
SPS B-D (Admin, prison officer, phys ed)	1010	*82.7*			
SPS E-G (manager, head of function, governor)	212	*17.3*			n/a
**Staff years worked in prisons**					
0-1	82	*(6.5)*			
2-4	197	*(15.7)*			
5-9	184	*(14.7)*			
10 or more	791	*(63.1)*			n/a

Supplementary Table 5: Bans factors (mean and standard deviation) and independent variables – CONTINUED

	Staff		Prisoners		
	**N**	***(%)***	**N**	***%***	**(Chi-sq sig)**
**AMONG PRISONERS ONLY**					
**Age left education**					
14 years or less			316	*(13.4)*	
15-16			1508	*(64.1)*	
17 or more			530	*(22.5)*	n/a
**Prisoner status**					
Unconvicted (awaiting trial)			411	*(17.5)*	
Convicted – release within 90 days			389	*(16.6)*	
Convicted – release 3-12 months			625	*(26.7)*	
Convicted – release over a year			918	*(39.2)*	n/a
**AMONG SMOKERS ONLY**					
**Cigarettes per day**					
10 or less	60	*(48.8)*	331	*(18.3)*	
11-20	53	*(43.1)*	1058	*(58.4)*	
21 or more	10	*(8.1)*	422	*(23.3)*	(<0.001)
**Craved cigarettes today**					
Not or hardly at all	42	*(34.4)*	281	*(15.4)*	
A little	37	*(30.3)*	416	*(22.7)*	
Somewhat, quite a bit or a great deal	43	*(35.2)*	1133	*(61.9)*	(<0.001)
**Quit attempt in (in prison) in past year**					
Yes	90	*(74.4)*	740	*(42.0)*	
No	31	*(25.6)*	1021	*(58.0)*	(<0.001)

© 2019 Sweeting H.
